# Yield gap analysis of rainfed alfalfa in the United States

**DOI:** 10.3389/fpls.2022.931403

**Published:** 2022-07-27

**Authors:** Rudra Baral, Romulo P. Lollato, Kamal Bhandari, Doohong Min

**Affiliations:** ^1^Department of Agronomy, Kansas State University, Manhattan, KS, United States; ^2^Department of Physics, Kansas State University, Manhattan, KS, United States

**Keywords:** Medicago sativa L., growing season rainfall, frontier yield function, linear boundary function, attainable yield, water-limited potential yield, conditional inference tree, yield gap

## Abstract

The United States (US) is the largest alfalfa (*Medicago sativa* L.) producer in the world. More than 44% of the US alfalfa is produced under rainfed conditions, although it requires a relatively high amount of water compared to major field crops. Considering that yield and production of rainfed alfalfa have been relatively stagnant in the country for decades, there is a need to better understand the magnitude of yield loss due to water limitation and how far from yield potential current yields are. In this context, the main objective of this study was to estimate the current yield gap of rainfed alfalfa in the US. We collected 10 year (2009–2018) county-level government-reported yield and weather data from 393 counties within 12 major US rainfed alfalfa producing states and delineated alfalfa growing season using probabilistic approaches based on temperature thresholds for crop development. We then calculated county-level growing season rainfall (GSR), which was plotted against county-level yield to determine attainable yield (Ya) using frontier function analysis, and water-limited potential yield (Yw) using boundary function analysis. Average and potential water use efficiencies (WUE) were estimated, and associated yield gap referring to attainable (YGa) or water-limited yields (YGw) were calculated. Finally, we used conditional inference trees (CIT) to identify major weather-related yield-limiting factors to alfalfa forage yield. The frontier model predicted a mean Ya of 9.6 ± 1.5 Mg ha^−1^ and an associated optimum GSR of 670 mm, resulting in a mean YGa of 34%. The boundary function suggested a mean Yw of 15.3 ± 3 Mg ha^−1^ at the mean GSR of 672 ± 153 mm, resulting in a mean yield gap of 58%. The potential alfalfa WUE was 30 kg ha^−1^ mm^−1^ with associated minimum water losses of 24% of mean GSR, which was three times greater than the mean WUE of 10 kg ha^−1^ mm^−1^. The CIT suggested that GSR and minimum temperature in the season were the main yield-limiting weather variables in rainfed alfalfa production in the US. Our study also revealed that alfalfa was only limited by water availability in 21% of the environments. Thus, future research on management practices to narrow yield gaps at current levels of water supply is necessary.

## Introduction

Alfalfa (*Medicago sativa* L.) is the fourth largest crop in terms of area harvested and production, and the third-largest crop in terms of economic value in the US (USDA-NASS, 2022). The country is also the largest alfalfa producer in the globe, with an annual hay production of above 50 million metric tons from about 7 million hectares which has a current market value of over 9 billion US dollars (Gardner and Putnam, 2018; USDA-NASS, 2022). Alfalfa is commercially cultivated in 42 states and several reasons justify such a widespread production, including its high nutritive value, high yield, long stand persistence, wide adaptation, being a perennial legume with biological nitrogen fixation, and soil benefits (Putnam et al., 2007; Adhikari and Missaoui, 2017). On average, alfalfa stands persist 4–5 years (average 4.5 years) in the US, depending on the crop management such as soil fertility, pests (weeds, insects, and plant diseases), weather (drought, severely cold temperature, and lack of snow cover).

The US domestic livestock production has increased by 19% over the past 10 years (USDA-NASS, 2021). Simultaneously, the export of livestock and meat products has increased by 21%, and of dairy products by 36%. Nonetheless, alfalfa area has declined by 18% and total production by 23%. Consequently, livestock feed and forage imports have increased by 25% (USDA-FAS, 2021). Although increased alfalfa production could help alleviate the need for hay importation, it is challenging to expand production area due to limited cropland, water scarcity, low yield in many states, subsidized competing crops (e.g., wheat, maize, soybean), lack of alfalfa insurance program, and expansion of maize acreage for ethanol production (Putnam et al., 2000; Creech and Foster, 2013). Furthermore, some evidence suggests that the maximum attainable yield of alfalfa is 2–3 times greater than the average on-farm yield which is mainly due to agronomic limitations and can be improved through improved drainage, appropriate variety selection, fertilizer management, cutting management, reduced traffic, improved stand establishment, weed and pest management, and irrigation efficiency (Putnam, 2021). In this context, quantifying potential forage yield, current forage yield gap, and its causes, can help to improve both yield and production and meet the growing forage demand. It can also help to guide research and development investments in regions with larger gaps (Edreira et al., 2017).

Water is the main factor limiting the yield of rainfed crops, including alfalfa (Tadesse et al., 2015; Holzman and Rivas, 2016). The water requirement of alfalfa is relatively high as compared to major cereal crops because it has a long growing season, deep root system, and a dense vegetation canopy (Takele and Kallenbach, 2001; Shewmaker et al., 2011; Schneekloth and Andales, 2017). Thus, growing season rainfall (GSR) and the water holding capacity of the soil are important modulators of rainfed alfalfa forage yield (Grimes et al., 1992; Jia et al., 2006; Jun et al., 2014). The dry matter yield of alfalfa is positively correlated with the amount of water used (Bauder et al., 1978; Shewmaker et al., 2011; Rogers et al., 2016), with a mean water-use efficiency (WUE, the ratio of crop biomass to evapotranspiration) of 16 kg ha^−1^ mm^−1^ (Lindenmayer et al., 2011). Jensen and Miller (1988) documented 155 to 213 mm of water is required to produce one metric ton of alfalfa forage whereas Shewmaker et al. (2011) reported 508 to 1,168 mm per season, depending on climate, elevation, growing season, number of cuttings, latitude, and alfalfa fall dormancy.

In the US, 24 states including the Midwest and some states of the South and Northeast regions, cultivate alfalfa mostly under rainfed conditions which accounts for over 44% of the US total area harvested (USDA-NASS, 2022). The mean annual alfalfa yield of those states ranges from 2.9 to 7.6 Mg ha^−1^ (USDA-NASS, 2022) whereas previous research conducted in some of these rainfed states found that yields over 13 Mg ha^−1^ are attainable (Table 1). Correspondingly, Russelle (2013) quantified the alfalfa yield gap in the US using several approaches ranging from 58 to 70% for non-irrigated regions, suggesting a large room for improvement in current yields. Beyond managerial factors, these rainfed US states have been experiencing moderate to exceptional drought for decades during the alfalfa growing season (April–October; Supplementary Figure 1). Takele and Kallenbach (2001) reported that moderate to severe drought can decline alfalfa yield up to 72%. Furthermore, genetic variation for drought tolerance alfalfa has not been thoroughly studied (Humphries et al., 2021; Kang et al., 2022). Hence, quantifying the level of water limitation to alfalfa yield grown under rainfed conditions in the US can help define regions where greater yield improvements are possible.

**Table 1:** Mean and range in alfalfa forage yield as reported by various field experiments during the study period (2009–2018).

State	Location	Yield (mean, range; Mg ha^−1^)	*n*	Source
Kansas	Manhattan, Topeka, Garden city	19.0 (21.3–16.1)	7	Kansas State University 2013; McDonald et al., 2021a,b
Kentucky	Lexington	13.7 (19.8–8.5)	10	University of Kentucky 2021
Minnesota	Dakota	15.6 (18.8–11.7)	10	University of Minnesota 2021
Nebraska	Havelock	19.1 (21.3–17.2)	8	University of Nebraska Lincoln 2016
New York	Ithaca	14.0 (15.4–12.5)	10	Cornell University 2021
North Dakota	Carrington	8.3 (12.6–5.1)	8	Wang et al., 2014; North Dakota State University 2018; Aponte et al., 2019
Ohio	South Charleston	14.2 (18.3–10.2)	10	Ohio State University 2019
Pennsylvania	Rock Spring	18.3 (24.6–13.8)	10	PennState Extension 2021
South Dakota	Beresford	15.4 (18.3–13.3)	5	Owens et al., 2017; Sara et al., 2019
Wisconsin	Outagamie	13.3 (14.7–12)	9	University of Wisconsin Madison 2016

*n* = Number of published field-based research data.

Similarly, temperature is another major abiotic factor affecting plant growth and yield. The growing season of summer and winter crops have been delineated as determined by the occurrence of minimum temperature (Tmin) thresholds (Purcell et al., 2003; Torres et al., 2013; Van Ittersum et al., 2013; Lollato et al., 2020). However, to our knowledge, there have been no attempts to objectively define the growing season for alfalfa across large growing regions. The available literature determines alfalfa growing season using very loose and arbitrary definitions, such as (i) a fixed day of the year (Raun et al., 1999), (ii) the occurrence of at least five days when the mean temperature is above 5°C (Sanderson et al., 1994), (iii) a flexible growing season definition based on a combination of the approaches above (Sulc et al., 1999), or (iv) the occurrence of Tmin below −2.8°C, a temperature threshold below which substantial damage to vegetative tissue occur in alfalfa (Sprague, 1955; Nath and Fisher, 1971; McKenzie and McLean, 1982). Despite the discrepancies above, there is a consensus that the base temperature for alfalfa is 5°C (Wolf and Blaser, 1972; Onstad and Fick, 1983; Fick et al., 1988; Sharratt et al., 1989; Confalonieri and Bechini, 2004), as seed germination and seedling growth are restricted below this threshold (Vough and Marten, 1971; Townsend and McGinnies, 1972; Andrews, 1987; Leep et al., 2001; Ahmed et al., 2019). Because determination of the length of the growing period is important to estimate crop potential yield (Purcell et al., 2003; Lobell et al., 2009; Torres et al., 2013), there is a need to objectively determine the growing season for alfalfa using daily weather data.

Several papers on yield gap analysis of major field crops are available such as wheat (Anderson, 2010; Neumann et al., 2010; Patrignani et al., 2014; Hatfield and Beres, 2019; Lollato et al., 2019), maize (Neumann et al., 2010; Grassini et al., 2011; Egli and Hatfield, 2014; Liu et al., 2017; Balboa et al., 2019), and soybean (Grassini et al., 2015a; Zhang et al., 2016; Balboa et al., 2019). However, to the best of our knowledge, an yield gap analysis of rainfed alfalfa in the US has not been performed and the magnitude of yield gap for these rainfed states is still unknown. Therefore, the overarching objective of this study was to estimate the yield gap of rainfed alfalfa in the major US rainfed alfalfa-growing regions. To do so, our specific objectives were to systematically (i) delineate county-specific alfalfa growing season using long-term weather data; (ii) calculate the cumulative growing season rainfall and optimum amount of rainfall required to obtain maximum yield; (iii) estimate minimum water losses (percolation, evaporation, runoff) and maximum WUE during the alfalfa growing season; (iv) compare maximum and attainable yields with the current yield at the state level, and (v) assess the possible weather determinants of alfalfa yield.

## Materials and methods

### Study area

**Figure 1:**
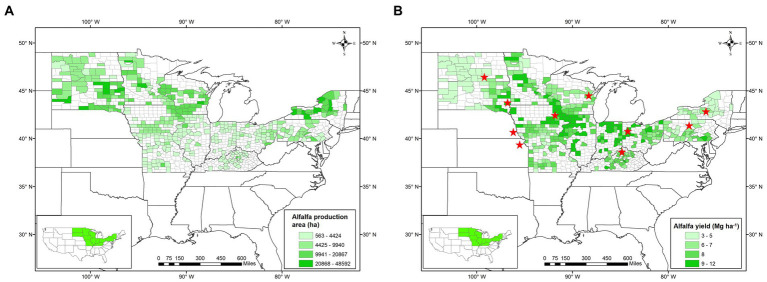
Map of the US showing selected rainfed alfalfa producing counties (*n* = 393) in green for harvested area **(A)** and forage yield **(B).** The red stars in panel B represent the locations (*n* = 10) from where alfalfa yield data obtained from research experiments were used for this study. Inset shows selected states as rainfed states within the continental US for this study.

Twenty-four states produce alfalfa mostly under rainfed conditions in the US (USDA-NASS, 2022; Supplementary Table 1). Out of those, 12 states (i.e., Illinois, Indiana, Iowa, Kentucky, Minnesota, Missouri, New York, North Dakota, Ohio, Pennsylvania, South Dakota, and Wisconsin) were selected for analyses based on their rainfed alfalfa production area and total production. In those states, more than 95% of the alfalfa is cultivated under rainfed conditions, thus, the selection of these states avoided significant inclusion of irrigated alfalfa fields in the database, as those are not reported separately. Those states represent different US growing regions, including the Southeast (Kentucky), Northeast (New York and Pennsylvania), upper Midwest (Iowa, Minnesota, North Dakota, South Dakota, Wisconsin), and lower Midwest states (Illinois, Indiana, Missouri, Ohio; Figure 1). We excluded the other 12 rainfed states because 11 states produce less than 1% of the US total rainfed alfalfa production and one state (i.e., Michigan) does not have county-level yield data.

For the 12 states above, county-level alfalfa yield was retrieved from USDA-NASS (2022) for the 2009–2018 period. The selected 393 counties from those 12 selected rainfed states accounted for more than 70% of the total rainfed alfalfa production in the US (Figure 1A), which is sufficient to reliably estimate yield gaps and the 10 year period avoids major technological trends (Van Ittersum et al., 2013). The soils in the study area are mainly Alfisols, Mollisols, and Entisols (Clark et al., 2019).

### Delineation of growing season

We developed code in Python 3.7.6 (Python Language Reference, version 3.7.6^1^ using information from Applied Climate Information System (ACIS) web services of National Oceanic and Atmospheric Administration-Regional Climate Centers (NOAA-RCCs) to obtain daily weather data of all stations located in each selected state for the 10 year period (2009–2018; ACIS, 2020). Then, we sorted the data by county using federal information processing system (FIPS) code, the number of digits that identify US state and county area (NOAA-NWS, 2022) and station name. For each of the 393 counties from 12 states (i.e., Illinois, Indiana, Iowa, Kentucky, Minnesota, Missouri, New York, North Dakota, Ohio, Pennsylvania, South Dakota and Wisconsin) included in the study, daily weather data for maximum and minimum temperatures, as well as rainfall, was selected from one weather station centrally located in the respective county. Counties with more than 10% missing weather data were interpolated from nearby ground stations. The days with missing values were replaced with the value of the nearest station located in the same county or adjacent county.

**Figure 2:**
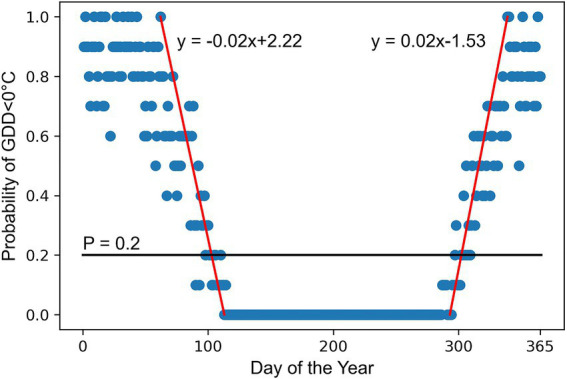
Methodology for estimating growing season duration for alfalfa using Noble County, Indiana, as an example. Solid black line represents 0.2 probability of occurrence of growing degree days (GDD) < 0°C using a base temperature of 5°C for each day of the year for the last 10  years. Solid red lines are the linear regression for decreasing (spring) and increasing (fall) probabilities of days where no GDD were accumulated.

The growing season of alfalfa is defined as the number of days in a year when the mean temperature is equal to or above the alfalfa’s base temperature, which is 5°C (Wolf and Blaser, 1972; Onstad and Fick, 1983; Fick et al., 1988; Sharratt et al., 1989; Confalonieri and Bechini, 2004). The selection of this temperature threshold is justified because alfalfa plants stop or significantly reduce their growth and development when temperatures fall below it. To delineate the alfalfa growing season, we used a method similar to that described for summer crops by Torres et al. (2013) and Purcell et al. (2003). Here, the temperature-limited alfalfa growing season for each county was estimated as the mean number of days between the last day in spring (called as growing season start day) and the first day in fall (called as growing season end day) when the probability of occurrence of growing degree days (GDD) < 0°C for each day of the year (DOY) for the 10 years considered in the analysis was 0.2 (*p* = 0.2; Figure 2). The 20% probability threshold is justified as there is a minimum effect on yield if exposed up to 2 days of Tmin <5°C out of 10 days. February 29 was ignored in leap years. The probabilities of GDD < 0°C were plotted against DOY and regressed separately against the decreasing probability values (between last day of *p* = 0 and first day of *p* = 100%) in the spring and the increasing *p* values (between last day of *p* = 100 and first day of *p* = 0%) in the fall as suggested by Torres et al. (2013). The resulting linear equations developed for each county were then used to calculate the respective DOY when *p* = 0.2. The mean of the county-level growing season length was considered as state-level growing season length.

After delineating the growing season length, we calculated growing season minimum and maximum temperature (Tmax), and cumulative GDD and rainfall. To understand the major weather drivers of alfalfa yield in the dataset, we created a conditional inference tree (CIT), which is a machine learning algorithm based on a regression model, to analyze the most significant weather variables contributing to alfalfa yield. The CIT described the conditional distribution of alfalfa yield predicted by multiple weather variables through tree-structured recursive partitioning (Hothorn et al., 2006). To avoid variable selection bias and overfitting, we performed (1) global null hypothesis test between any of the randomly selected weather variables and alfalfa yield and then selected the best predicting weather variable which had the lowest root mean square error (RMSE) and highest R^2^ values, (2) binary split on the selected predicting variables, and (3) recursively repeated step 1 and 2. We started with a CIT that allowed a minimum of 5% of the observations to be included in intermediate nodes and of 1% of observations to be included in the terminal nodes. We then increased these bucket sizes to allow for the inclusion of minimums of 40 and 10%. The model fit exercise selected the most parsimonious CIT that resulted in changes of less than 5% in R^2^ from the initial CIT. The CIT was built in R using the package partykit (Hothorn et al., 2015).

### Yield and yield gap estimation

We used recorded alfalfa hay yield data combined with weather records of 393 counties from 2009 to 2018 for the estimation of three types of yield: (i) current yield (Yc), (ii) attainable yield (Ya), and (iii) water-limited potential yield (Yw). The yield gap (Yg) was then calculated as the difference between Yw and Yc or Ya and Yc. The WUE was assessed using linear boundary function approach used by French and Schultz (1984).

The Yc was calculated as the weighted mean yield of the last 10 years at a county level, assumedly using adapted alfalfa cultivars grown under the existing production environment and management practices.


(1) $$Y_{c}=\overset{n}{\underset{i=1}{\sum }}\frac{\;Y_{\mathrm{wt}}}{N}$$

Where *Y*_c_ is the state-level current yield, *Y*_wt_ is the weighted yield of alfalfa of county 𝑖 and n is the number of year.

**Figure 3:**
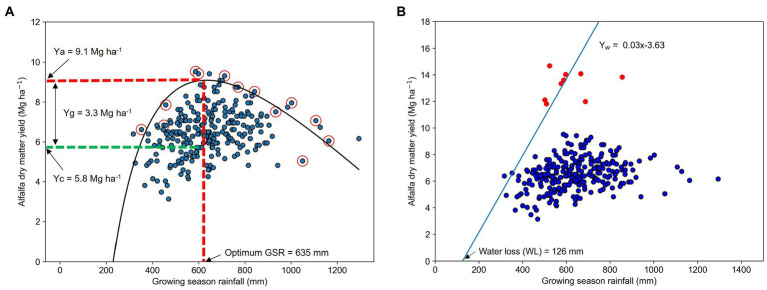
Example of estimating **(A)** attainable yield (Ya) using frontier yield function and **(B)** water-limited potential yield (Yw) using linear boundary function, in this example for the state of Wisconsin (WI). The value in the peak of the nonlinear line in panel **A** represents maximum attainable yield (the red dotted line intersecting *Y*-axis) at an optimum growing season rainfall of 635 mm (the red dotted line intersecting in *X*-axis). The slope of a linear line in panel **B** represents the water-limited potential yield (Yw) obtained from growing season rainfall, after allowing for a minimum loss of 126 mm during the entire growing season. The blue dotted markers represent annual yield for a 10 year period (2009–2018) of selected counties within WI (*n* = 29) obtained from USDA-NASS. The red circled dotted markers in panel A represent maximum yield achieved in a county within a certain rainfall range and the red dotted points in panel B represent data from variety trials conducted at Outagamie County of WI during the study period.

We used Cobb–Douglas stochastic frontier yield function to estimate Ya (Neumann et al., 2010; Figure 3A). In this approach, the Ya was calculated as the maximum yield of alfalfa hay that was ever achieved within a county in the last 10 years at a given level of GSR. The entire range in GSR was divided into equally spaced bins, and the maximum yield from each bin was selected. Then those selected values were plotted against corresponding GSR and regressed using nonlinear regression (Neumann et al., 2010; Patrignani et al., 2014).


(2) $$\ln Y_{a}=\beta_{0}+\beta_{1}\ln X_{i}+\beta_{2}\left(\ln X_{i}^{2}\right),\mathrm{where}\;X_{i}>0$$

where $Y_{a}$ is the attainable yield which represents the maximum yield ever achieved by county i within last 10 years at each rainfall bin, $X_{i}$ represents the corresponding GSR of county i , $\beta_{0}$ is the intercept when yield is zero, and $\beta_{1}$ and $\beta_{2}$ are the slope of parameters of GSR. The maximum yield value found on the frontier line is referred as the Ya and the corresponding rainfall as optimum rainfall to achieve maximum yield for the given county. The optimum GSR at which the yield is maximized was determined by equating derivative of frontier yield with respect to GSR to zero, and solving the equation for this particular rainfall amount returns the maximum yield value. The quadratic nature of this function is justified as Ya is expected to decline at lower or greater rainfall amounts due to insufficient or excessive water supply or harvesting losses due to continuous or heavy rainfall during harvesting time.

Water-limited potential yield (Yw) was estimated using linear boundary function (Figure 3B) following French and Schultz (1984) approach. Here, county-level yield data were plotted against corresponding GSR, dividing the GSR range in which yield is responsive to increases in water supply into equally spaced bins, selecting the maximum yield within each rainfall bin, and regressing both variables. The resulting linear equations developed from this model are used to estimate state-level Yw.


(3) $$Y_{w}=WUE\;\left(GSR-WL\right)$$

Where $Y_{w}$ represents theoretical water-limited potential yield, the slope of the linear regression is WUE, and WL is the minimum non-productive water losses. The difference between GSR and WL is the amount of water used by the crop during the entire growing season to obtain maximum potential yield. To ensure the robustness of the linear function and to increase the range in yields for linear function development, we added variety trial data of each state similar to the approach by Patrignani et al. (2014). Variety trial data of the study period was obtained from the respective state’s extension reports and published research papers (Table 1; Figure 1B).

## Results

### Growing season delineation

The length of the alfalfa growing season for the selected rainfed states ranged from 169 days in Minnesota to 241 days in Kentucky, with a mean of 204 growing days in a year (Table 2). The growing season was longer in the lower Midwest and Southeast regions (206 to 241 days), where it started from mid-March (73 DOY in Kentucky) to early April (100 DOY in Iowa) and ended in the second week of November (316 DOY in Missouri). This compared with 169–192 days of growing season duration in the upper Midwest and Northeast regions, where it started from the second to the third week of April (107 DOY in Wisconsin to 113 DOY in Minnesota) through the second to last week of October (282 DOY in Minnesota to 300 DOY in Wisconsin).

**Table 2:** Estimated alfalfa growing season for 12 rainfed states including start and end day in day of year (DOY), and key weather variables.

State	Growing season						
	Start DOY	End DOY	Total GDD	Tmax (°C)	Tmin (°C)	Tav (°C)	Accumulated GDD (^o^C-day)
Illinois	91 (31-Mar)	309 (4-Nov)	218 ± 16	23.9 ± 0.7	12 ± 0.9	18 ± 0.7	2,783 ± 274
Indiana	94 (3-Apr)	311 (6-Nov)	217 ± 11	23.8 ± 0.8	11.9 ± 1.2	17.9 ± 0.9	2,779 ± 251
Iowa	100 (8-Apr)	306 (31-Oct)	206 ± 10	24.1 ± 0.8	11.9 ± 1.0	18.0 ± 0.8	2,574 ± 216
Kentucky	73 (12-Mar)	314 (9-Nov)	241 ± 24	24.9 ± 1	12.8 ± 1.1	18.8 ± 1	3,278 ± 313
Minnesota	113 (22-Apr)	282 (8-Oct)	169 ± 20	22.8 ± 0.7	10.7 ± 1.1	16.8 ± 0.8	1,964 ± 244
Missouri	82 (22-Mar)	316 (10-Nov)	234 ± 19	24.8 ± 0.7	12.6 ± 0.8	18.7 ± 0.7	3,152 ± 242
New York	109 (17-Apr)	298 (23-Oct)	189 ± 12	22.5 ± 0.9	10.6 ± 1	16.6 ± 0.7	2,156 ± 253
North Dakota	115 (23-Apr)	284 (10-Oct)	170 ± 19	23.4 ± 0.9	9.8 ± 1.2	16.6 ± 1	1,935 ± 142
Ohio	92 (31-Mar)	307 (1-Nov)	21 ± 10	23.8 ± 0.8	11.7 ± 0.8	17.8 ± 0.7	2,692 ± 191
Pennsylvania	94 (3-Apr)	306 (31-Oct)	212 ± 13	23.6 ± 0.7	11.5 ± 1.4	17.6 ± 0.9	2,624 ± 314
South Dakota	109 (17-Apr)	294 (19-Oct)	185 ± 21	24.2 ± 0.8	10.7 ± 1.2	17.5 ± 0.8	2,268 ± 211
Wisconsin	107 (16-Apr)	300 (25-Oct)	193 ± 14	22.1 ± 1.1	10.5 ± 1.1	16.3 ± 0.8	2,155 ± 210
**Mean**	**98 (7-Apr)**	**302 (28-Oct)**	**204** ± **22**	**24** ± **0.8**	**110** ± 0**.9**	**18** ± **0.8**	**2,530** ± **422**

Weather variables are 10 year’s mean maximum and minimum temperature and accumulated GDD during the alfalfa growing season across the selected counties.

### Growing season temperature and rainfall

**Figure 4:**
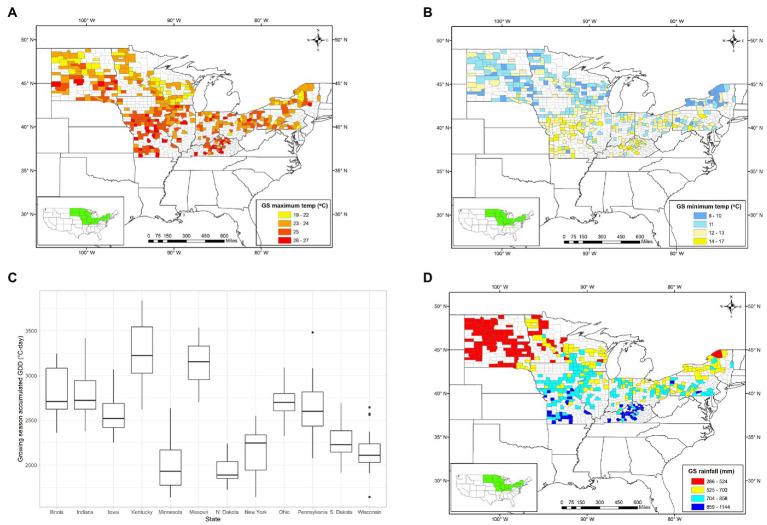
Mean growing season maximum **(A)** and minimum **(B)** temperature, accumulated growing degree day **(C)** and cumulative growing season rainfall **(D)** in the study area during the 2009–2018 period.

Mean growing season temperature increased from the north (16.7± 0.4°C) to the south (18.1 ± 0.5°C). The maximum mean temperature during the growing season was 23 ± 0.7°C in upper Midwest and Northeast states and 24.1 ± 0.5°C in lower Midwest and Southeast states, whereas the minimum mean temperature was 10.4 ± 0.4°C in upper Midwest and Northeast states and 12.1 ± 0.4°C in lower Midwest and Southeast states (Figures 4A,B; Table 2). The accumulated GDD during the growing season was 2,096 ± 126°C-day for the upper Midwest and Northeast regions and was 2,840 ± 249°C-day for the lower Midwest and Southeast regions, with North Dakota showing the lowest (1,964°C-day) and Kentucky showing the highest accumulated GDD (3,278°C-day; Figure 4C; Table 2).

Over the 2009–2018 period, the mean annual rainfall within the study area ranged from 285 mm in Sioux County (ND) to 1,143 mm in Hardin County (KY), with a mean of 672 mm (ACIS, 2020). The mean GSR in the upper Midwest and Northeast was estimated as 525 ± 101 mm. The GSR in Wisconsin has slightly higher (648 ± 66 mm) than those in other upper Midwest states. Similarly, the GSR in the lower Midwest, Southeast, and Northeast states was estimated at 778 ± 77 mm. North Dakota was the driest state, followed by South Dakota, and Minnesota whereas the highest rainfall was recorded in Kentucky, followed by Missouri and Illinois (Figure 4D; Table 3). Moreover, the optimum GSR required to produce the maximum possible yield in the study region was estimated 670 ± 113 mm.

**Table 3:** State-level GSR optimum GSR, minimum water loss, mean WUE, potential WUE and percentage of GSR below the optimum requirement.

State	Mean GSR (mm)	Optimum GSR (mm)	Minimum water loss	Minimum water loss (%)	Mean WUE (kg ha^−1^ mm^−1^)	Potential WUE (kg ha^−1^ mm^−1^)	% of GSR below the optimum
Illinois	746 ± 56	759	190	25	10	27	27
Indiana	740 ± 69	768	171	23	10	27	26
Iowa	745 ± 45	698	176	24	10	31	17
Kentucky	936 ± 92	880	214	23	8	27	21
Minnesota	542 ± 119	630	77	14	13	34	22
Missouri	827 ± 87	719	195	24	7	27	12
New York	632 ± 59	587	154	24	8	34	14
North Dakota	366 ± 40	391	75	21	12	27	13
Ohio	693 ± 56	645	215	31	11	38	22
Pennsylvania	743 ± 67	636	253	34	8	31	19
South Dakota	441 ± 77	688	109	25	13	34	47
Wisconsin	648 ± 66	635	126	19	10	27	15
Mean	672 ± 153	670 ± 113	163 ± 54	24 ± 5	10 ± 2	30 ± 4	21 ± 9

### Alfalfa yield, potential water use efficiency, and yield gap

The current alfalfa hay yield at the state level ranged from 4.8 to 7.8 Mg ha^−1^ and averaged 6.4 ± 1 Mg ha^−1^ (Table 4), though at the county level Yc ranged from 2.7 Mg ha^−1^ in Perkins County of South Dakota to 10.8 Mg ha^−1^ in Clinton County of Illinois (Figure 1A). The state of Iowa had the highest Yc of 7.8 ± 1 Mg ha^−1^ with a mean GSR of 698 mm. Meanwhile, New York and North Dakota had the lowest Yc (4.8 ± 1 Mg ha^−1^) which might be partially due to a concomitant lowest mean GSR at least in North Dakota (366 mm). Our frontier yield model predicted an alfalfa Ya of 9.6 Mg ha^−1^ at an optimum GSR of 670 mm, which is almost 51% greater than the Yc and originated a mean attainable yield gap of 34%. The highest alfalfa Ya was 11.9 Mg ha^−1^ and occurred in Indiana, with an optimum GSR of 768 mm. Our linear model resulted in alfalfa WUE of 30 kg ha^−1^ mm^−1^, with minimum water losses (x-intercept in Figure 3B) of 163 mm (or 24% of mean GSR). Meanwhile, mean WUE was 10 kg ha^−1^ mm^−1^, representing a significant WUE gap. Also, on average 21% of the GSR was found below the optimum GSR required to obtain maximum yield, suggesting that water availability does not limit alfalfa yields in this region in 79% of the cases in the study regions (Table 3). The linear boundary function estimated a mean alfalfa Yw of 15.3 Mg ha^−1^ at the mean GSR of 672 mm, which is 140% greater than the Yc and originated a mean yield gap of 58%. The highest estimated Yw was 19.4 Mg ha^−1^ at a mean GSR of 880 mm in Kentucky whereas the lowest Yw was 7.8 Mg ha^−1^ at a mean GSR of 366 mm in North Dakota.

**Table 4:** State-level current yield (Yc), attainable yield (Ya), water-limited potential yield (Yw) and yield gap in comparison to attainable (YGa) and water-limited yield (YGw).

State	Yc (Mg ha^−1^)	Ya (Mg ha^−1^)	Yw (Mg ha^−1^)	YGa (Mg ha^−1^)	YGw (Mg ha^−1^)	YGa (% of Ya)	YGw (% of Yw)
Illinois	7.6	11.5	15.5	3.9	7.8	34	51
Indiana	7.5	11.9	15.0	4.4	7.4	37	50
Iowa	7.8	11.0	17.9	3.2	10.1	29	57
Kentucky	6.7	9.2	19.4	2.5	12.8	28	66
Minnesota	7.0	10.5	15.6	3.5	8.6	33	55
Missouri	5.6	8.7	17.0	3.1	11.4	36	67
New York	4.8	6.9	16.1	2.1	11.3	31	70
North Dakota	4.8	7.4	7.8	2.6	3.0	35	39
Ohio	7.2	10.1	18.2	2.9	11.1	29	61
Pennsylvania	5.9	8.5	15.4	2.7	9.5	31	62
South Dakota	5.8	10.9	11.2	5.1	5.4	47	48
Wisconsin	5.8	9.1	14.1	3.3	8.3	36	59
Mean	6.4 ± 1.0	9.6 ± 1.5	15.3 ± 3	3.3 ± 0.8	8.9 ± 2.6	34 ± 5	58 ± 9

### Weather variables driving alfalfa yield

**Figure 5:**
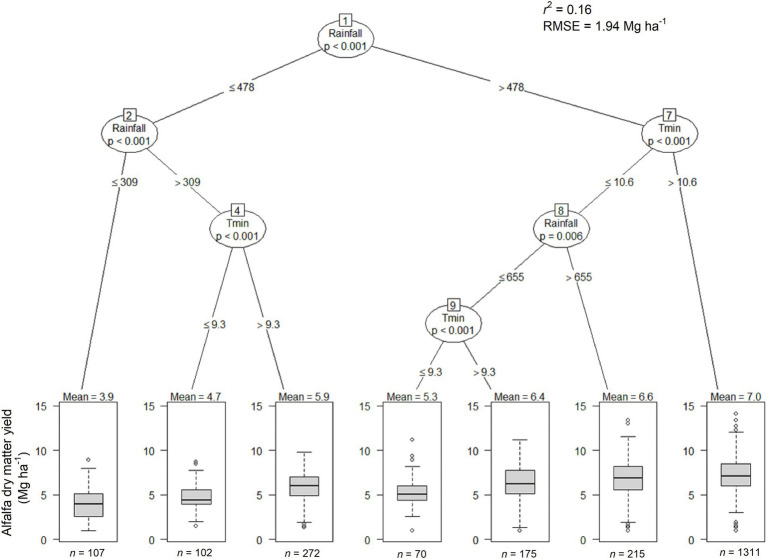
Conditional inference tree for alfalfa forage yield as function of the interaction between growing season weather variables (cumulative rainfall and growing degree days, and mean Tmax and Tmin) for 393 counties over 10  years period. The oval-shaped circle contains a specific predictor that affects yield, the branches represent binary partition of the variables based on the shown threshold of predictor. Each boxplot inside the terminal nodes represents the interquartile range (gray box), median (solid line inside the gray box), upper and lower extreme (whiskers), and outliers (black circles) of alfalfa yield in Mg ha^−1^. The value of n on the bottom of the terminal node represents the total number of observations in each terminal node. The GSR is expressed in mm and Tmin in ^o^C.

The most parsimonious CIT explaining alfalfa yield as determined by weather variables resulted in an *R^2^* of 0.16 and a *RMSE* of 1.94 Mg ha^−1^ and suggested that GSR and Tmin were the largest drivers of alfalfa yield (Figure 5). The highest yields (~7 Mg ha^−1^) occurred in seasons and areas with GSR > 478 mm and Tmin >10.6°C. In cooler seasons (Tmin <10.6°C), alfalfa yields ranged from 5.3 to 6.6 Mg ha^−1^ depending on temperature and rainfall regimes. For growing seasons receiving between 309 and 478 mm, alfalfa yields depended on Tmin with cooler seasons (<9.3°C) yielding less than warmer seasons (4.9 vs. 5.9 Mg ha^−1^). The lowest yields (3.9 Mg ha^−1^) were associated with GSR <309 mm.

## Discussion

We used a systematic approach previously used for summer crops (Purcell et al., 2003; Torres et al., 2013) to delineate the duration of alfalfa growing season representing over 70% of the total rainfed alfalfa area in the US. Subsequently, we calculated weather variables for the growing season that allowed for calculating attainable yield and water-limited yield based on GSR, benchmarking WUE for the calculation of yield gaps. Lastly, we identified major weather drivers of rainfed alfalfa yield in this large geography.

An original contribution of this research is that, to our knowledge, this is the first systematic delineation of growing season duration using a probabilistic approach for a perennial crop such as alfalfa, which to date has been performed arbitrarily and inconsistent (Sprague, 1955; Nath and Fisher, 1971; McKenzie and McLean, 1982; Sanderson et al., 1994; Raun et al., 1999; Sulc et al., 1999). The duration of the growing seasons using the current method aligned well with the alfalfa growing season recommended by respective states’ forage agronomists for the lower Midwest and Southeast regions (Henning and Nelson, 1993; Lacefield et al., 1997; Stachler, 2016; Rocateli et al., 2017), as well as for the upper Midwest and Northeast regions (Undersander et al., 2015; Wells et al., 2018). From a practical standpoint, the estimated start DOY in spring and end DOY in the fall could be considered as the cropping calendar for optimum planting and last cutting dates for the respective states. More broadly, this is an important contribution to perennial crop systems in general as we showed that the method here presented could potentially be used to delineate the growing season for other perennial crops in other regions.

We used 10 year of weather and yield data to estimate Yc, Ya and Yw, which is sufficient for relevant representation of the system and avoids possible effects of changes in production factors such as advancement of production technology, expansion of irrigation program, breeding improvement or climate change effect on crop yield (Van Wart et al., 2013; Grassini et al., 2015b). Furthermore, the government reported county-level yield data was only available after 2009. Thus, considering the need for a minimum of 10 year of yield data, this is the first time when such analysis is possible at a country level for alfalfa.

Several methods have been used to estimate crop yield gaps, such as remote sensing (Dehkordi et al., 2020; Wang et al., 2020), crop model simulation (Jones et al., 2003; Yang et al., 2004; Lobell and Ortiz-Monasterio, 2006; Van Wart et al., 2013; Lollato et al., 2017; Soltani et al., 2020; Jaenisch et al., 2021; Jáuregui et al., 2022), upper percentile of farmer’s yield (Laborte et al., 2012; Affholder et al., 2013; Van Ittersum et al., 2013), field experiment or yield contest (Lobell et al., 2009; Van Ittersum et al., 2013; Lollato et al., 2019), maximum farmers yield (Lobell et al., 2005, 2009; Van Ittersum et al., 2013), boundary-function analysis (Lobell et al., 2009; Affholder et al., 2013; Van Ittersum et al., 2013; Patrignani et al., 2014; Sadras et al., 2015), linear boundary function (French and Schultz, 1984; Milne et al., 2006; Sadras and Angus, 2006; Patrignani et al., 2014; Sadras et al., 2015) and frontier yield function model (French and Schultz, 1984; Lobell et al., 2009; Soltani and Sinclair, 2012; Affholder et al., 2013; Van Ittersum et al., 2013; Patrignani et al., 2014). Here, we used two approaches (i.e., frontier yield and linear boundary functions) that consider the biophysical boundaries of crop yield potential determination in rainfed systems based on GSR. These methods allow for the respective estimation of attainable yield and water-limited potential yield, which are reasonable approaches to estimate Yg in rainfed systems (Van Ittersum et al., 2013).

Our study revealed that there is a large Yg in rainfed alfalfa in the US as current yields are only 66% of the attainable yield and 42% of the water-limited potential yield. Our results are consistent with many previous studies. For example, Russelle (2013) reported up to 70% Yg in non-irrigated alfalfa regions in the US Similarly, Soltani et al. (2020) estimated 69% Yg in Iran and Jáuregui et al. (2022) estimated 53% in Argentina under rainfed conditions. Our estimated Yw seems to be similar to the yield obtained from the variety trials or research experiments conducted in the study area and nearby locations (Table 1). Thus, the estimated region-specific Yw based on the WUE benchmark we developed in this manuscript can be used as a benchmark alfalfa yield under rainfed conditions (Van Ittersum et al., 2013; Sadras et al., 2015). Meanwhile, the decrease in Ya below and above the estimated optimum GSR (Figure 3A) is likely due to water deficit stress in the low end (Takele and Kallenbach, 2001; Djaman et al., 2020; Qiu et al., 2021), and either excessive water regarding the crop requirements during the growing season (Graven et al., 1965; Thompson and Fick, 1981), waterlogging issues that may decrease biological N fixation (Havelka et al., 1982; Andrés et al., 2012), or increase harvest losses due to continuous or heavy rainfall during harvesting and drying time.

The large yield gap found for rainfed alfalfa in the US using the current approaches suggests that water availability is usually not a limiting factor to alfalfa yield in the region. Correspondingly, only 21% of the environments had total GSR less than the optimum GSR required for maximum forage yield, and the resulting WUE was about three times less than the potential WUE (Table 3). This suggests that, at least in c.a., 80% of the environments, alfalfa yields are limited by factors other than water availability, e.g., distribution of growing season rainfall or crop management factors. While the goal of the current manuscript was not to determine best management practices for reduced alfalfa yield gaps, we highlighted the opportunity to improve alfalfa yields at current levels of water supply. Future research should focus on strategies to minimize the current gap at the farm level.

In rainfed production systems, evapotranspiration and WUE have linear relationship with alfalfa forage yield (Lindenmayer et al., 2011; Undersander et al., 2015; Li and Su, 2017). Our model predicted that the most efficient use of growing season rainfall by alfalfa resulted in about 24% minimum water losses with the remaining 76% mostly being used by plants through transpiration with a 30 kg ha^−1^ mm^−1^ efficiency (Table 3). While the fractions of seasonal water available and lost were within the range reported by previous studies (Kool et al., 2014; Li et al., 2017; Berg and Sheffield, 2019; Wagle et al., 2021), the WUE (27–36 kg ha^−1^ mm^−1^) was greater than it the majority of the other studies—as expected due to the nature of the boundary function. Still, this slope is similar to that reported by Fink (2021) in a global analysis of alfalfa maximum WUE; and careful evaluation of the data reported by Lindenmayer et al. (2008) suggests a few maximum WUE values in the same range, validating our linear model. Furthermore, we found higher WUE and lower dry matter yield in the upper Midwest and Northeast states as these states have dryer and cooler summer (Tables 2 and 3). These results correspond well with previous studies conducted by Lindenmayer et al. (2008), Ismail and Almarshadi (2013), and Li et al. (2017) who also reported greater WUE and lower dry matter yield under water-stressed conditions. The reason behind higher WUE could be the ability of alfalfa to extract moisture from deeper soil layers and to remain dormant during moisture-stressed conditions. Meanwhile, the lower yield could be the result of insufficient GSR, well below the values required to produce maximum attainable yield (Table 3).

While we found a wide range of yield variability across the rainfed states which associated with the variability in weather conditions, we note that weather conditions only explained about 16% of yield variability of alfalfa. This low explanatory power could be a function of the coarse scale of this study, evaluating a single reference weather station centered at the county—which may not be representative of weather conditions occurring in other parts of the county. Likewise, the current approach does not consider rainfall distribution, thus, while the total amount may not be limiting, distribution may be. Nonetheless, within the limitations of our analysis, data suggested that GSR and Tmin were the main drivers of alfalfa productivity at the county level. These results are supported by numerous previous studies (Boyer, 1982; Takele and Kallenbach, 2001; Zeid and Shedeed, 2006; Lafitte et al., 2007; Hamidi and Safarnejad, 2010; Thivierge et al., 2016; Ren et al., 2021) suggesting that water stress can adversely affect plant growth, reproductive capacity, yield, quality, and alfalfa survival. Similarly, a recent study by Pourshirazi et al. (2022) estimated up to 33% alfalfa yield loss due to cold temperatures in Iran. In alfalfa, the production of interest is predominantly biomass. Biomass accumulation is driven by solar radiation but modulated by moisture and temperature regimes (Soltani and Sinclair, 2012); therefore, the effect of GSR and Tmin on alfalfa forage yield is straightforward. Furthermore, lower Tmin could lead to slow regrowth (Evenson, 1979; Körner, 2015) resulting in fewer cuttings per season, which associate negatively with alfalfa annual yield (Fink, 2021).

## Conclusion

Our study estimated the current yield gap of 34% when compared to attainable yield, and of 58% when compared to the water-limited potential yield from the evaluation of about 70% of the rainfed alfalfa production area in the US. These results revealed that in most of the environments evaluated, alfalfa yield was only limited by suboptimal water availability in 21% of the cases. Our results suggested that GSR and Tmin were two key weather factors associated with alfalfa forage yield. We found higher attainable yield and water-limited potential yield in lower Midwest and Southeast regions as these states have warmer summer, longer growing season, and higher growing season rainfall than those in the upper Midwest and Northeast regions.

The findings of this study are particularly useful for alfalfa forage growers to realize that their production is mostly not limited by water availability and thus indicating that they could manage their production resources (i.e., labor, capital, technology, and irrigation) for higher yield levels for the given amount of water supply. Likewise, these results should be considered by government officials and policymakers to develop research and development programs that can address the current alfalfa forage yield gap. Of interest to other major perennial crops, this study also provides a reliable method to calculate the growing season based on probabilities of growing degree day accumulation, which allows for estimates of growing season weather variables needed to estimate yield gaps.

## Data availability statement

Publicly available datasets were analyzed in this study. This data can be found at: National Oceanic and Atmospheric Administration, Regional Climate Centers (http://www.rcc-acis.org/docs_webservices.html#title19). Data and Statistics. United States Department of Agriculture, National Agricultural Statistics Service (https://www.nass.usda.gov/Data_and_Statistics/index.php).

## Author contributions

RB prepared the manuscript, analyzed data, and developed maps and Figures. RL conceptualized the study, designed the methodology, and reviewed the manuscript. KB developed Python code for data analysis, maps and Figures. DM attained funding for project conduction and reviewed the manuscript. All authors contributed to the article and approved the submitted version.

## Funding

This project was partially supported by the United States Department of Agriculture, National Institute of Food and Agriculture (USDA-NIFA) Competitive grant no. 2019-70005-30238, Grant no. 2019-68012-29888, and Grant no. USDA-NIFA-OP-006691(2-562460.KSU).

## Conflict of interest

The authors declare that the research was conducted in the absence of any commercial or financial relationships that could be construed as a potential conflict of interest.

## Publisher’s note

All claims expressed in this article are solely those of the authors and do not necessarily represent those of their affiliated organizations, or those of the publisher, the editors and the reviewers. Any product that may be evaluated in this article, or claim that may be made by its manufacturer, is not guaranteed or endorsed by the publisher.

## Footnotes

^1^ http://www.python.org

## Supplementary material

The Supplementary material for this article can be found online at: https://www.frontiersin.org/articles/10.3389/fpls. 2022.931403/full#supplementary-material

SUPPLEMENTARY FIGURE 1 Percent area of the Midwest region of the US affected by drought from 2000 to 2020 during alfalfa-growing period (April to October). The severity of the drought is categorized as D0: Abnormally dry, D1: Moderate drought, D2: Severe drought, D3: Extreme drought, and D4: Exceptional drought (NDMC, 2021).
