# Supplementary figures and images for: Yield gap analysis of rainfed alfalfa in the United States
Source: Front Plant Sci. 2022 Jul 27;13:931403. doi: 10.3389/fpls.2022.931403 (PMC9363835; doi:10.3389/fpls.2022.931403)

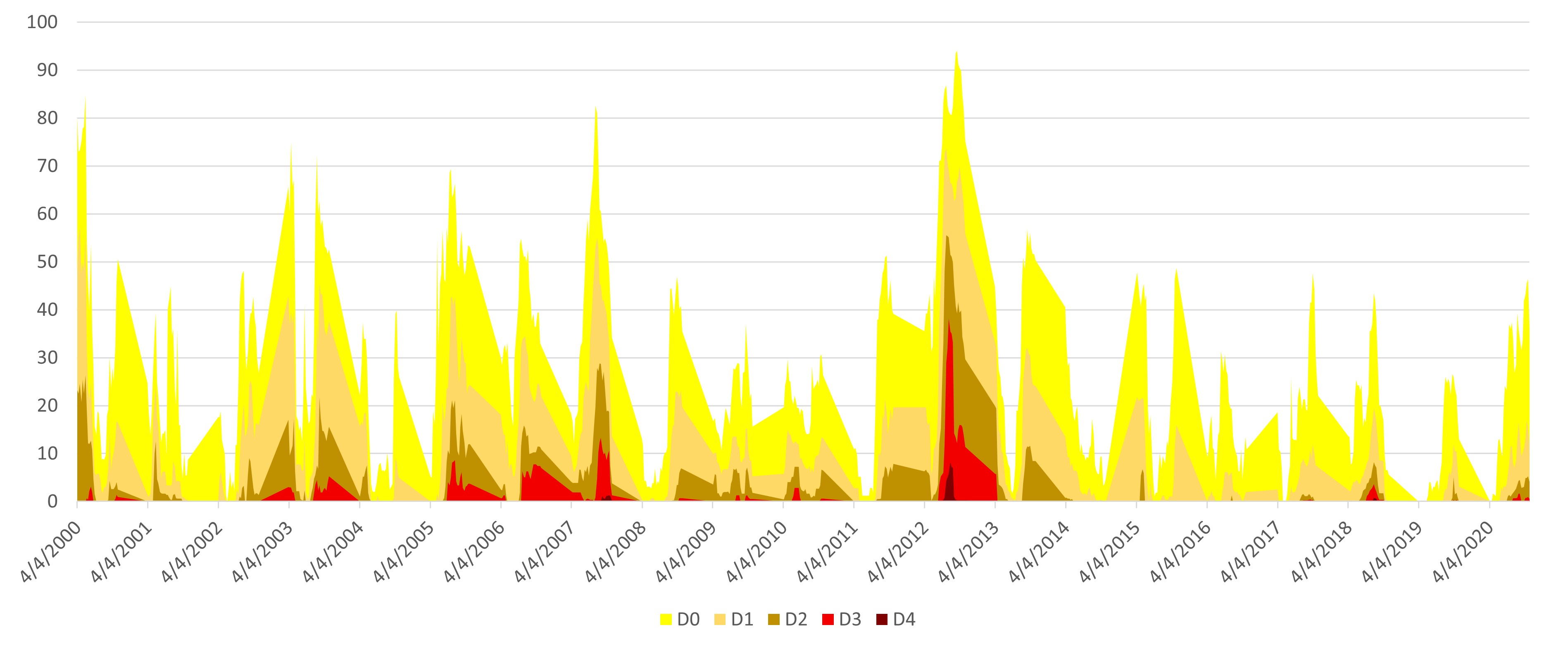

Supplement: SUPPLEMENTARY FIGURE 1 — Percent area of the Midwest region of the US affected by drought from 2000 to 2020 during alfalfa-growing period (April to October). The severity of the drought is categorized as D0: Abnormally dry, D1: Moderate drought, D2: Severe drought, D3: Extreme drought, and D4: Exceptional drought (NDMC, 2021). [file Image_1.JPEG]
