# Supplementary material for: Yield gap analysis of rainfed alfalfa in the United States
Source: Front Plant Sci. 2022 Jul 27;13:931403. doi: 10.3389/fpls.2022.931403 (PMC9363835; doi:10.3389/fpls.2022.931403)
Supplement: Supplementary file 2 [file Table_1.DOCX]

**Supplemental Table 1.** Selection of States based on alfalfa production and rainfed production area

| State | Irrigated area (ha) | Irrigated % | Rainfed % | Total harvested area (ha) | % US harvested area | Production (tons) | % of US total production | % of US total rainfed production |
| --- | --- | --- | --- | --- | --- | --- | --- | --- |
| SOUTH DAKOTA | 32175 | 4.4 | 95.6 | 729374 | 9.0 | 2877067 | 4.5 | 6.5 |
| NORTH DAKOTA | 111 | 1.0 | 99.0 | 5943 | 8.1 | 2089778 | 3.3 | 4.7 |
| WISCONSIN | 6218 | 0.8 | 99.2 | 335459 | 5.2 | 3071064 | 4.8 | 7.0 |
| MINNESOTA | 3524 | 1.9 | 98.1 | 233267 | 4.1 | 2345020 | 3.6 | 5.3 |
| IOWA | 140 | 0.1 | 99.9 | 11716 | 3.5 | 2385316 | 3.7 | 5.4 |
| MICHIGAN | 1051 | 1.5 | 98.5 | 91997 | 2.9 | 1351628 | 2.1 | 3.1 |
| OHIO | 6396 | 0.2 | 99.8 | 659201 | 2.1 | 1217582 | 1.9 | 2.8 |
| PENNSYLVANIA | 3325 | 0.2 | 99.8 | 421689 | 2.0 | 1161432 | 1.8 | 2.6 |
| NEW YORK | 214 | 0.2 | 99.8 | 91233 | 1.8 | 836233 | 1.3 | 1.9 |
| ILLINOIS | 346 | 0.1 | 99.9 | 148154 | 1.2 | 845430 | 1.3 | 1.9 |
| INDIANA | 362 | 1.1 | 98.9 | 166859 | 1.1 | 696575 | 1.1 | 1.6 |
| MISSOURI | 298 | 0.2 | 99.8 | 166015 | 1.1 | 554878 | 0.9 | 1.3 |
| KENTUCKY | 142 | 0.0 | 100.0 | 100430 | 1.0 | 587809 | 0.9 | 1.3 |
| VIRGINIA | 269 | 0.0 | 100.0 | 285409 | 0.4 | 209188 | 0.3 | 0.5 |
| MARYLAND | 0 | 1.2 | 98.8 | 3190 | 0.1 | 96179 | 0.1 | 0.2 |
| VERMONT | 0 | 0.0 | 100.0 | 77903 | 0.1 | 46278 | 0.1 | 0.1 |
| NEW JERSEY | 0 | 1.9 | 98.1 | 3547 | 0.1 | 40604 | 0.1 | 0.1 |
| WEST VIRGINIA | 0 | 0.0 | 100.0 | 3545 | 0.1 | 34818 | 0.1 | 0.1 |
| NORTH CAROLINA | 0 | 0.0 | 100.0 | 1088 | 0.1 | 29842 | 0.0 | 0.1 |
| TENNESSEE | 0 | 0.0 | 100.0 | 5505 | 0.1 | 28473 | 0.0 | 0.1 |
| MAINE | 0 | 0.0 | 100.0 | 4560 | 0.0 | 14576 | 0.0 | 0.0 |
| MASSACHUSETTS | 0 | 0.0 | 100.0 | 10416 | 0.0 | 20973 | 0.0 | 0.0 |
| CONNECTICUT | 0 | 0.0 | 100.0 | 32243 | 0.0 | 13542 | 0.0 | 0.0 |
| NEW HAMPSHIRE | 0 | 0.0 | 100.0 | 5912 | 0.0 | 5241 | 0.0 | 0.0 |

*Note: The top 12 states are selected for this study. Because of the unavailability of county-level alfalfa yield data, we excluded Michigan State for this study.*
